# Peer role-play and standardised patients in communication training: a comparative study on the student perspective on acceptability, realism, and perceived effect

**DOI:** 10.1186/1472-6920-10-27

**Published:** 2010-03-31

**Authors:** Hans M Bosse, Martin Nickel, Sören Huwendiek, Jana Jünger, Jobst H Schultz, Christoph Nikendei

**Affiliations:** 1 Clinic I General Paediatrics, Centre of Child and Adolescent Medicine, Heidelberg, Germany; 2 Department of Psychosomatic and General Internal Medicine, University of Heidelberg Medical Hospital, Germany

## Abstract

**Background:**

To assess the student perspective on acceptability, realism, and perceived effect of communication training with peer role play (RP) and standardised patients (SP).

**Methods:**

69 prefinal year students from a large German medical faculty were randomly assigned to one of two groups receiving communication training with RP (N = 34) or SP (N = 35) in the course of their paediatric rotation. In both groups, training addressed major medical and communication problems encountered in the exploration and counselling of parents of sick children. Acceptability and realism of the training as well as perceived effects and applicability for future parent-physician encounters were assessed using six-point Likert scales.

**Results:**

Both forms of training were highly accepted (RP 5.32 ± .41, SP 5.51 ± .44, n.s.; 6 = very good, 1 = very poor) and perceived to be highly realistic (RP 5.60 ± .38, SP 5.53 ± .36, n.s.; 6 = highly realistic, 1 = unrealistic). Regarding perceived effects, participation was seen to be significantly more worthwhile in the SP group (RP 5.17 ± .37, SP 5.50 ± .43; p < .003; 6 = totally agree, 1 = don't agree at all). Both training methods were perceived as useful for training communication skills (RP 5.01 ± .68, SP 5.34 ± .47; 6 = totally agree; 1 = don't agree at all) and were considered to be moderately applicable for future parent-physician encounters (RP 4.29 ± 1.08, SP 5.00 ± .89; 6 = well prepared, 1 = unprepared), with usefulness and applicability both being rated higher in the SP group (p < .032 and p < .009).

**Conclusions:**

RP and SP represent comparably valuable tools for the training of specific communication skills from the student perspective. Both provide highly realistic training scenarios and warrant inclusion in medical curricula. Given the expense of SP, deciding which method to employ should be carefully weighed up. From the perspective of the students in our study, SP were seen as a more useful and more applicable tool than RP. We discuss the potential of RP to foster a greater empathic appreciation of the patient perspective.

## Background

In order to establish a functional patient-physician relationship, physicians are required to sense the individual reality of the patient [1]. While the underlying clinician-patient communication processes are complex and poorly understood, the quality of these processes can predict health outcomes even months after consultations have taken place [2]. "Good communication skills in medical practice are not innate, can be learned, and can always be enhanced" [3]. Investing in resources to improve physician communication skills is therefore worthwhile in terms of enhancing patient adherence [3,4].

Training communication skills requires professional practice. For the successful enhancement of communication skills, it is well established that medical educators should use experimental rather than purely didactic methods [5] in order to enable acquired skills to be integrated into further clinical practice [6-8]. Several such methods are available and include, for example, peer role-play and the use of standardised patients. A number of studies have shown that all experimental methods require additional measures of consolidation, such as workshops with standardized patients [9] or continuous clinical supervision [10], in order to achieve a sustainable transfer of the skills acquired in training to clinical work.

Standardised patient (SP) is an umbrella term used to refer to simulated patients (trained to simulate patient illnesses) as well as actual patients (trained to present their own illness); both of whom present their symptoms in a standardised manner [11,12]. In line with this definition, the term SP is used in the current study to refer to simulated patients. SP are classified as high-technology instruments and are an expensive tool for the training of communication skills [13]. They provide a high degree of realism and have considerable potential for effectively training general and specific communication skills [6,12,14,15]. They are also suitable for both formative and summative assessments of communication skills [16,17]. The professional feedback provided by SP is a key to their success [17,18]. In the field of paediatrics, SP may be integrated into the curriculum in the form of paediatric standardised patients [19] or - as is the case in the present publication - as standardised parents [20,21].

Peer role-play (RP) is a low-cost tool which is relatively easy to install. It allows trainees to experience the perspective of both the physician and the patient. Experiencing these multiple perspectives and the ambiguity of the partners involved in communication [22,23] helps to improve trainees' understanding of the complexity of the physician-patient interaction. If RP training-sessions are carefully designed and tutors well-trained, initial scepticism regarding participation in RP may be resolved [24].

Both SP and RP are frequently employed in the teaching of communication skills worldwide [25]. The majority of previous studies have shown RP or SP to be superior to no intervention as well as to purely didactic methods including lectures [26-28], oral instructions [29], or educational materials such as a clinical manual [30]. In their comprehensive review, Lane & Rollnick [25], however, identified major methodological weaknesses in almost all studies conducted so far. These weaknesses pertained to a lack of randomisation, small sample sizes, potential biases (providing no baseline assessments), the instruments used (validity, reliability, internal consistency), and the fact that results were based on dichotomous assessments of communication skills rather than the quality of a specific performance. As a result of these methodological weaknesses, drawing definitive conclusions on the specific effects of RP and SP is difficult.

Moreover, given the expense associated with using standardised patients, clarifying the *differential value *of the two methods is essential. As far as we are aware, only three studies have so far compared SP and RP [31-33]. In the first study conducted by Papdakis et al. [33], undergraduates rated SP training more favourably than RP. Following training with RP or SP, each student was assessed based on a single encounter with an SP. No performance differences were found between the two groups. In a second randomised, controlled trial, Mounsey et al. [32] also failed to find a significant difference between RP and SP based on the quality of videotaped interviews which were conducted by undergraduates following the respective interventions and which were assessed using a validated instrument. In a similar randomised, controlled study design, Lane et al. [31] trained health-care professionals using either a fellow trainee or a SP. Following training, each health professional performed one interview with one SP. This interview was rated using a validated measure of practitioner skill in behaviour-change counselling. Both groups showed the same post-training level of competence and there were no significant group differences with respect to the associated affect or applicability of the sessions as rated by the health care professionals. In summary, the two tools have been found to be of comparable effectiveness in motivational interviewing and have been shown to result in similar levels of skill attainment in both undergraduates and health professionals. However, all three studies are limited to one specific challenge in communication - that of facing addiction as one specific communicational challenge. In our opinion, the conclusions of these studies therefore can not be generalized to a broader medical context.

We conclude that experimental settings are undoubtedly required for effective communication training, and several studies have emphasized the advantages which RP or SP may carry for communication training. Presently, it is difficult to draw conclusions regarding the specific value of RP and SP in communication training in a broader medical context. In our randomised study, we therefore compared undergraduates' views on the value of RP and SP in the framework of a broader range of medical issues and challenges in communication.

The research questions examined in the present study concerned students' ratings of the two methods with respect to acceptability and the realism of scenarios; students' ratings of the training methods with respect to both methods being worthwhile, useful, and applicable for future exploration and counselling of parents; and whether students' ratings of the two methods significantly differed. Given the methodological additional expense involved in SP, we assumed that training with SP would be better accepted by the students, be considered to provide more realistic scenarios, and be perceived as more effective.

## Methods

### Training cases

Major medical problems and major communication problems in our paediatric outpatient department were defined by an expert group using a focus group approach as previously described by our study group [21]. Nine training cases were developed which combined the nine most common medical and communication problems (see Table 1). Detailed and specific learning objectives were defined for both the medical (including exploration and counselling) and the communication problems. Cases were subsequently designed in such a way as to be appropriate for training with RP or SP and taking all predefined learning objectives into account, maintaining the same complexity across both groups. This included designing concise patient and physician briefing sheets for the RP group and concise physician briefing sheets and detailed SP scripts for the SP group.

**Table 1:** Training cases

Case	Medical problem	Communication problem
Case 2	Urticaria	Dramatizing mother
Case 3	Diarrhea	Foreign mother with poor command of German
Case 4	Abdominal pain	Conflict due to long waiting times
Case 5	Fever	Demanding mother
Case 6	Crying baby	Anxious and overburdened mother
Case 7	Meningism	Parents disapprove of drug administration
Case 8	Febrile seizure	Parents reject lumbar puncture of the child
Case 9	Dyspnea	Parents oppose admission of the child

Nine training cases were designed combining a medical and a communication problem.

### Sample

#### Randomisation procedure, allocation concealment, and blinding

Students of the Medical Faculty of Heidelberg who were in their prefinal (i.e., fifth) year and eligible for their four-week rotation in paediatrics (N = 69) participated in the study and were randomly assigned to one of the two consecutively conducted study groups. The first group received communication training employing peer role-play (RP group, N = 34) and the second group received communication training with standardised patients (SP group, N = 35). Both forms of training took place in addition to established course contents which were identical across study groups for the four-week rotation and included seminars, problem-based learning, computer-based training using CAMPUS [34], bedside teaching, and placements in paediatric private practices [35]. Allocation concealment or blinding was not possible due to the nature of the course and the study design. Due to the fact that Heidelberg students opt for rotations abroad and may do so at short notice, there was a drop-out rate of N = 3 (8.8%) in the RP group and N = 2 (5.7%) in the SP group, resulting in study groups of N = 31 (RP group) and N = 33 (SP group). Students of both study groups had had extensive previous experience with both RP and SP in their medical courses prior to their paediatrics rotation.

#### Sex, age, semester, and study motivation

Prior to the interventions, students were asked to complete questionnaires regarding their *sex*, *age*, *semester*, and *study motivation *(six-point Likert scale ranging from 6 = *very high *to 1 = *very low*). The questionnaire return rate was 96.7% in the RP group and 96.9% in the SP group.

#### Pre-intervention communication competence

Communication competence was assessed based on the mean self-rating of 24 positive statements which covered 14 general communication skills and 10 specific communication skills as specified in the learning goals defined prior to the intervention. The rate of return for questionnaires was 96.7% in the RP group and 96.9% in the SP group.

### Tutors

Twelve physicians and psychologists (male N = 6, female N = 6) with more than three years of experience in student communication training served as tutors in the training sessions. All tutors received the same training prior to the study. Tutors were provided with a manual containing precise instructions and briefing sheets for each of the cases.

### Standardised patients

Actors with more than two years of experience as SP received professional training based on the scripts at the SP training-centre of our medical faculty. Their performance was assessed and approved by experienced paediatricians prior to the intervention.

### Training sessions including feedback

Within each of the study groups, students were assigned to small groups comprising three students. Each small group was trained in a total of three training sessions over the course of three consecutive weeks with one tutor per small group. Training in the SP group additionally included one trained SP per small group and per case. The effective time students spent in training was held constant across both groups: Training sessions in both experimental groups lasted two and a half hours and consisted of three training cases in which the students rotated in their role as *physician*, *parent*, and *observer *in the RP group or as *physician *and *observer *in the SP group. These rotations allowed each student to assume an active role in each training session as has previously been recommended [36]. Before commencing the interview, students in the *parent *role in the RP group were allowed to take as much time as necessary to go through the instructions. In our study, students required between 4 and 10 minutes to do so.

Following a briefing by the tutor, the student in the physician role conducted a 10-minute interview and subsequently reflected on his/her performance. This was followed by feedback from the student in the parent role (RP group) or structured feedback from the standardised patient in the parent role (SP group). Observers subsequently provided structured feedback using a checklist addressing the predefined major medical and interaction issues of the respective case. Finally, the tutor provided concluding structured feedback which was followed by a time of group discussion and debriefing [17,37].

To control for potential contamination between the study groups, a total of three one-hour seminars covering the key medical issues addressed in the scenarios were offered parallel to the three weeks of training and were attended by an average of 90.3% (RP group) and 90.9% (SP group) of the students.

### Outcomes

Outcomes assessed in both study groups included the student perspective on a) acceptance of the training, as assessed by the *overall grade for the training *(six-point Likert scale ranging from 6 = *very good *to 1 = *very poor*), b) realism of cases ("*The cases were realistic*"), and perceived effects of the training, as assessed by ratings of c) the time spent in training having been worthwhile ("*Taking part in the intervention was worthwhile*") and d) the usefulness of the training ("*The intervention was useful for training communication skills*"; six-point Likert scale ranging from 6 = *totally agree *to 1 = *don't agree at all*). All outcomes were assessed after each of the three training sessions with an average questionnaire return rate of 90.3% in the RP group and 90.9% in the SP group. Subject to additional assessment after the three training sessions was e) the applicability for future exploration and counselling of parents of sick children ("*I feel well prepared for exploration and counselling of parents of sick children*"; six-point Likert scale ranging from 6 = *totally agree *to 1 = *don't agree at all*) with a questionnaire return rate of 90.3% in the RP group and 87.8% in the SP group.

### Power calculation

To achieve a power of .9 with a two-sided significance level of 5% for a minimum effect size of .5 with respect to a) the *overall grade *for the training, b) the *realism of cases*, c) the *training being worthwhile*, d) the *usefulness*, and e) *applicability *of the training, and a standard deviation of .5, a required sample size of 23 was calculated. Based on the actual sample sizes and given results, a post-hoc two-tailed test of power yielded .93, .64, and .87 for the *training being worthwhile*, the *usefulness*, and *applicability *of the training, respectively.

### Statistical analysis

Samples were compared using a least significant difference test and outcomes were assessed using a Student's t-test for independent variables.

Research was carried out in compliance with the Helsinki Declaration. In light of the above described study design, the University of Heidelberg Ethics Committee waived requirements for an ethical approval procedure.

## Results

### Sample

#### Sex, age, semester, and study motivation

No significant group differences were found with respect to *sex*, *semester*, or *study motivation*. However, a small difference was found with respect to *age *(RP 23.7 ± .7 years, SP 25.5 ± 3.0 years, p < .002; Table 2).

**Table 2:** Consistency of the two study groups

Items	Peer role-play (RP) group	Standardized patient (SP) group	P
Male	19 (61.3%)	15 (45.5%)	n.s.
Female	11 (35.5%)	16 (48.5%)	n.s.
Sex not specified	1 (3.2%)	2 (6.0%)	-
Age	23.7 ± .7	25.5 ± 3.0	< .002

Semester	9.2 ± .7	9.3 ± .8	n.s.

Motivation	5.1 ± .6	4.9 ± .9	n.s.

Self-assessment of communication skills	6.20 ± 1.02	6.51 ± 1.41	n.s.

To demonstrate consistency of the two study groups, the distribution of sex, age, semester, study motivation, and pre-intervention self-assessment of communication skills with parents of sick children are presented for each group (RP group N = 31, SP group N = 33). Age in years, semester in number of semesters of medical study, study motivation ranging from 6 = very high to 1 = very low, self-assessment of communication with parents of sick children as overall score of specific and general communication skills using a 10-point Likert scale ranging from 10 = totally agree to 1 = don't agree at all. Values are stated as means and standard deviations (SD) or N and percentage (%), including level of significance (p) calculated using the least significant difference test.

#### Pre-intervention communication competence

There was no significant group difference in mean pre-intervention communication competence (6.20 ± 1.02 for RP group, 6.51 ± 1.41 for SP group, n.s.; 10-point Likert scale ranging from 10 = *completely agree *to 1 = *don't agree at all*; Table 2).

### Acceptability

The *overall grade *for the training sessions was very high in both groups (5.32 ± .41 for RP group, 5.51 ± .44 for SP group; 6 = very good, 1 = very poor), with no significant difference between the two groups (Table 3).

**Table 3:** Assessment of the training

	Group	Mean	SD	p
Overall grade for the training	Peer role-play	5.32	.41	<.104
	Standardized patient	5.51	.44	

The cases were realistic.	Peer role-play	5.60	.38	<.478
	Standardized patient	5.53	.36	

Taking part in the intervention was worthwhile.	Peer role-play	5.17	.37	**<.003**
	Standardized patient	5.50	.43	

The intervention was useful for training communication skills.	Peer role-play	5.01	.68	**<.032**
	Standardized patient	5.34	.47	

I feel well prepared for exploration and counseling of parents of sick children.	Peer role-play	4.29	1.08	**<.009**
	Standardized patient	5.00	.89	

Assessment of training with respect to acceptability, realism, and effect in the peer-role-play group and the standardized-patient group stated as means and standard deviations (SD), with 6 = very good and 1 = very poor for the overall grade and 6 = totally agree and 1 = don't agree at all for all other items. Significance (p) using an unpaired Students t-test.

### Realism of training cases

The training cases were seen as *realistic *in both groups (5.60 ± .38 for RP group, 5.53 ± .36 for SP group; 6 = totally agree, 1 = don't agree at all), with no significant difference between the two groups (Table 3).

### Perceived effect of training

The time spent in training was seen as *worthwhile *by both groups (5.17 ± .37 for RP group, 5.50 ± .43 for SP group; 6 = totally agree, 1 = don't agree at all), with a significantly higher rating being reported in the SP group (p < .003). Both groups found the training cases to be very *useful in training communication skills *(5.01 ± .68 for RP group, 5.34 ± .47 for SP group; 6 = totally agree, 1 = don't agree at all), with the SP group again reporting significantly greater usefulness (p < .032). With respect to training *applicability*, both groups felt moderately *well prepared for future exploration and counselling of parents of sick children *after the training (4.29 ± 1.08 for RP group, 5.0 ± .89 for SP group; 6 = totally agree, 1 = don't agree at all), with significantly higher applicability being reported in the SP group (p < .009, Table 3).

## Discussion

We present a randomized, controlled study in which the student perspective on communication training with peer role-play was compared with that of training with standardised patients. From the student perspective, peer role-play and standardised patients appear to represent comparably valuable tools for undergraduate communication training: Both methods were very well accepted and perceived to be highly realistic. Both methods were also seen as worthwhile, useful, and applicable in training student communication skills. Great care was taken to demonstrate consistency of the two groups prior to the intervention, and in particular no differences were found with respect to *self assessed skills in communication*. A small but statistically significant *age difference *was not considered relevant to the research questions.

### Acceptance

We found a high degree of training acceptance in both study groups. While high acceptance has previously been demonstrated for SP [17,38] and was therefore expected, it was found to be surprisingly high in our peer role-play group, given that fewer resources are required for this method. Tackling difficult yet relevant problems - as was the case in our training [38] - has been shown to be essential for the acceptance and success of peer role-play in both postgraduate settings [39-41] and undergraduate curricula [36]. In each of the presented cases, we combined a relevant medical task with a relevant communication problem; a fact which may further have contributed to the acceptance of our cases [38]. A further factor contributing to the equal levels of high training acceptance found in both study groups was that the cases were of identical structure and equally high complexity [22] across groups and that tasks corresponded with students' level of prior experience and their curriculum [42]. We assume that the observed high acceptance also reflects students' enjoyment of taking on an active role and observing other people playing a role - be it an SP or a peer. These positive affects found in connection with both methods are considered essential when it comes to benefiting from learning opportunities, as has previously been demonstrated for role-play [36].

### Realism of training cases

Training scenarios were seen as highly realistic in both the SP and peer role-play group. This is in line with previous findings of our study group on SP training among health professionals [37,38]. A high degree of challenge and critical-decision moments was offered in the cases we employed in both study groups [21,37] as a prerequisite for high ratings of case realism [36,43]. An additional important factor for realistic scenarios which was also incorporated in our study is the prior training of tutors, who help students round off the role-play scenarios in their imagination [44,45].

Often seen as the ultimate method for simulating patients [13], the employment of standardised patients may have been expected to result in a high degree of perceived realism. However, in contrast to our expectations, the peer role-play scenarios - which comprised identical medical and communication issues but which were methodologically less elaborate - were rated as being equally realistic. The identical structure and equally high complexity of scenarios in addition to well-trained tutors appears to make up for the potential methodological advantage of SP with respect to perceived realism. A high degree of realism in training scenarios is a prerequisite for successful implementation of role play [42] and is particularly useful for overcoming the initial resistance often seen among participants engaging in peer role-play [24,42,46].

### Perceived effect of training

Both groups reported that the training cases were useful and worthwhile, and that they offered a moderately high degree of applicability for future exploration and counselling of parents of sick children. All of these aspects of perceived effectiveness were rated significantly higher in the standardised-patient group. Since the amount of training time, the qualification and presence of the tutors, the degree of scenario complexity, and students' overall ratings for the training were equal across the groups, the higher applicability in the SP group may well be ascribed to the specific form of training itself. Training with standardised patients was not new to the students - both study groups had had extensive exposure to standardised patients in courses prior to the present study.

One aspect which may explain the higher ratings of perceived effectiveness in the SP group is the value of the professional feedback provided by standardised patients [12,43,47] - a central factor supporting the individual learning process [47]. Comments (feedback) from the group constitute a key element of role play, as well as in the training with standardised patients [12,22,36,42,48,49]. Our standardised patients were specifically trained to focus on professionally structuring and phrasing aspects of the patient's inner perspective. This may provide more multifaceted feedback than that provided by peers, who potentially place greater emphasis on clinical issues when offering feedback. A further aspect is that students see SP as a high-tech simulation instrument [11-13] which they may expect to be more useful and applicable. Alternatively, students may simply enjoy watching a professional actor and may thus rate perceived training effects to be higher than those for RP.

Although SP may be associated with a higher perceived effect and applicability in communication training, only peer role-play allows trainees to experience the inner perspective of the patient and the ambiguity of the communication situation [23], yielding a deeper understanding of patients' concerns as well as greater empathy [50]. To ensure such changes in communication culture, communication training must encourage trainees to take heed of the perceptions of patients and their families [51], thus enhancing their perceptual skills within the communication process rather than simply relying on practicing behavioural skills [52]. We conclude that both peer role-play and standardised patients warrant inclusion in medical curricula: Peer role-play may allow students to personally experience patients' concerns, whereas standardised patients may have more potential with respect to training specific communication skills.

### Standardised parents

*Standardised parents *are used in a postgraduate setting for the training of counselling skills [53], breaking bad news [20,54], and for training communication in common paediatric problem situations as previously reported by our study group [21,37,55]. Feedback from standardised parents has been shown to be acceptable and useful in a postgraduate setting [37]. Among the plethora of data on standardised patients, there has, to our knowledge, been only one study published on the use of standardised parents in undergraduate training, with faculty members in the roles of parents [56]. This study showed an improvement in history-taking skills, although baseline skills were not assessed prior to training. Our findings underscore the value of standardised parents as an acceptable, realistic, and useful tool for implementation in undergraduate communication training.

### Limitations

#### Effect of training

We examined students' perceptions of peer role-play and standardised patients but did not investigate students' objective performance or the specific methodological impact of the two methods on variables influencing intermediate and major health outcomes [2]. Furthermore, our findings cannot be generalized to postgraduates and/or health professionals.

#### Study design

While the study was conducted in a controlled, randomised design, groups commenced consecutively owing to the structure of the four-week paediatric course with cohorts of 30 to 40 paediatric-rotation students at a time. Systematic errors resulting from the consecutive start (the first group being trained with peer role-play, the second with standardised patients) were controlled for through randomisation, and we were able to demonstrate good consistency between the two study groups with respect to socioeconomic data, motivation, and pre-intervention communication skills.

#### Applicability

We found significantly higher perceptions of applicability in the standardised-patient group, although we did not include tools to promote sustainable change in communication - such as consolidation workshops with standardised patients [9] or continuous clinical supervision [10] - in either of the groups.

### Further research

Future research should compare the effects of the two methods on students' objective performance, i.e. using MiniCEX or OSCE, based on standardised patients or real patient encounters. Future research should also address the long-term impact of both training methods. The effect of peer role-play on sustainable changes in communication has so far not been subject to investigation, as a result of which the differential and specific contributions of the two methods to the sustainability of accomplished goals remains unclear. Beyond these intermediate outcomes, future research should be guided by the model presented by Street et al. [2], examining the specific methodological impact of the two training forms on variables influencing major health outcomes.

## Conclusions

From the student perspective, peer role-play and standardised patients represent comparably valuable tools for undergraduate communication training. The positive affects towards to both methods represent an essential prerequisite for benefitting from both learning opportunities. Peer role-play as less elaborate tool than standardised patients offers just as highly realistic training scenarios, provided that great care is taken in designing the training.

Communication training with standardised patients is perceived as a slightly more useful tool than peer role-play. Furthermore, it potentially has a higher degree of applicability which both may be due to professional feedback provided by standardised patients as one of the main effectors of individual progress in skills trainings. On the other hand, the ambiguity offered by peer role-play suggests a methodological advantage in fostering an appreciation of patient concerns in addition to the development of skills.

Both peer role-play and standardised patients warrant inclusion in medical curricula: peer role-play constitutes a valuable additional tool for undergraduate communication training, requiring few resources and allowing students to personally experience patient concerns, whereas standardised patients potentially have a greater effect on specific communication skills. Given the expense, time and resources standardised patients require, peer role-play may be a good alternative. Deciding which of the two methods to employ should be carefully weighed up.

## Competing interests

The authors declare that they have no competing interests.

## Authors' contributions

All authors substantially contributed to the conception and planning of study as well as to the drafting of the manuscript. All authors read and approved the final manuscript. HMB coordinated the expert groups which defined learning goals of the training as well as developing the cases, the screen plays for the standardised patients, and the briefing sheets. CN, JHS, and HMB structured and organised the training sessions. HMB held the seminars relating to medical issues addressed in the training. SH assessed and approved the performance of the standardised patients. JJ is in charge of the standardised patient training centre at the Medical Faculty of Heidelberg and provided the resources to train the standardised patients. CN and JHS trained the tutors for the training sessions. MN designed the assessment forms and organised the assessment. CN, MN, and HMB performed the statistical analysis.

## Pre-publication history

The pre-publication history for this paper can be accessed here:

http://www.biomedcentral.com/1472-6920/10/27/prepub
